# Astragaloside IV Inhibits Bleomycin-Induced Ferroptosis in Human Umbilical Vein Endothelial Cells by Mediating LPC

**DOI:** 10.1155/2021/6241242

**Published:** 2021-11-01

**Authors:** Shuai Sheng, Jialin Xu, Qingyang Liang, Lei Hong, Li Zhang

**Affiliations:** ^1^Department of Cardiology, The First Affiliated Hospital of Guangdong Pharmaceutical University, Guangzhou, China; ^2^Department of Cardiology, Long Gang Central Hospital of Shenzhen, Shenzhen, China

## Abstract

Ferroptosis, as an iron-dependent programmed cell death pathway, can induce a variety of cardiovascular diseases. Astragaloside IV (AS-IV), which is purified from *Astragalus membranaceus*, can protect endothelial function and promote vascular regeneration. However, the role played by AS-IV in ferroptosis remains unknown. In this study, the lipid metabolomics in HUVECs treated with/without bleomycin and/or AS-IV were explored using LC/MS. The most differential metabolite between groups was further identified via GO and pathway enrichment analyses. The effects of lysophosphatidylcholine (LPC), AS-IV, and FIN56 on cell viability were explored using the CCK-8 assay, their effects on cell senescence were examined by *β*-galactosidase staining, and their effects on ferroptosis were detected by a flow cytometric analysis of lipid ROS levels, transmission electron microscopy, and an assay for cellular iron levels. The related mechanisms were investigated by real-time PCR and Western blot assays. Our results showed that LPC, as the most differential metabolite, inhibited cell viability but promoted cell apoptosis and senescence as its concentration increased. Also, the decreased cell activity, increased iron ion and lipid ROS levels, and the enhanced cell senescence induced by LPC treatment were all significantly reversed by AS-IV but further enhanced by FIN56 treatment. The changes in mitochondrial morphology caused by the LPC treatment were significantly alleviated by the AS-IV treatment, while treatment with FIN56 reversed those phenomena. Moreover, AS-IV partially upregulated the levels of SLC7A11 and GPX4 expression which were reduced by LPC. However, those changes were prevented by FIN56 treatment. In conclusion, our data suggested that AS-IV could serve as a novel drug for treating ferroptosis-related diseases.

## 1. Introduction

Vascular endothelial cells, as important components of arterial intima, do not only form the barrier between blood and tissues but also regulate blood vessel function and maintain a stable internal environment [1, 2]. When endothelial cells are continuously damaged, vascular pathological changes are often induced that make them susceptible to further damage caused by peroxidation. This additional damage leads to thickening of the vessel wall and lumen narrowing [3], which promotes thrombosis and vascular necrosis [2, 4]. Vascular cell senescence often induces vascular aging, which can lead to a variety of cardiovascular disorders such as atherosclerotic plaque formation, myocardial infarction, and heart failure [5, 6]. Lee et al. [7] suggested that lysophosphatidylcholine (LPC) is closely related to the vascular inflammation that occurs during senescence. Furthermore, bleomycin was also found to induce cell senescence [3]. Although some drugs, including statins and blockers/inhibitors of the renin-angiotensin-aldosterone system, can improve endothelial dysfunction, the rates of cardiovascular disease morbidity and mortality remain high [8, 9]. Therefore, it is still important to develop new drugs for treating and preventing cardiovascular metabolic diseases.

Recently, numerous reports revealed that ferroptosis is an iron-dependent oxidative and nonapoptotic cell death pathway [10]. The main morphological features of ferroptosis are a decreased mitochondrial volume, reduced or absent mitochondrial crest, ruptured mitochondrial outer membrane, but a normal-sized nucleus, and no nuclear concentration, all of which are the main morphological features of ferroptosis that distinguish it from apoptosis, necrosis, and autophagy [11]. An accumulation of lipid peroxides, and especially phospholipid peroxides, is considered to be the hallmark event of ferroptosis [12]. Many studies have found that ferroptosis is related to the progression of nervous system diseases, cardiovascular and cerebrovascular diseases, cancer, and other diseases. It has also been proposed that induction or inhibition of ferroptosis might be a new strategy for treating those diseases [13, 14]. Some natural products, most of which are polyphenols, have been found to inhibit ferroptosis by various mechanisms. For example, gelatin induces GPX4, FPN1, and ACSL4 expression via the Nrf2-HMOX1 signaling pathway and thereby inhibits glutamate-induced ferroptosis in HT-22 cells [15]. However, there are no reports concerning natural compounds that can treat cardiovascular metabolic diseases by inhibiting ferroptosis.

AS-IV, as a natural compound extracted and purified from *Astragalus membranaceus*, was found to function as an antioxidant molecule, inhibit myocardial hypertrophy, protect endothelial function, and promote vascular regeneration [16–18]. Studies demonstrated that Astragaloside IV protects the endothelial function by reducing ROS production and oxidative stress [19, 20]. Xu et al. [21] found that AS-IV can protect HUVECs from H_2_O_2_-induced oxidative stress by inhibiting the NADPH oxidase-ROS-NF-*κ*B pathway and eNOS uncoupling [21]. Although AS-IV has been studied for the treatment of cardiovascular disease, the effect of AS-IV on the ferroptosis of endothelial cells remains unknown.

In this study, differential metabolites of AS-IV in bleomycin-treated endothelial cells were screened in a lipid metabolomic analysis, and LPC was selected for further study. The most suitable concentration of LPC was selected after determining its inhibitory effects on cells; after which, the effects of LPC on cell senescence and damage were investigated. The influence of AS-IV on LPC-treated HUVEC cells was also explored. A ferroptosis agonist (FIN56) was used to further confirm the role of AS-IV in the ferroptosis of endothelial cells. Our findings suggested new targets and strategies for treating ferroptosis-related diseases.

## 2. Materials and Methods

### 2.1. Cell Culture and Treatment

Human umbilical vein endothelial cells (HUVECs) were purchased from ATCC (Manassas, VA, USA) and cultured in F-12K Medium (Thermo Fisher Scientific, Waltham, MA, USA) supplemented with 10% FBS (GIBCO, Grand Island, NY, USA), 0.1 mg/mL heparin (Selleck Chemicals, Houston, TX, USA), and 30 *μ*g/mL Endothelial Cell Growth Supplement (Thermo Fisher) at 37°C in a 5% CO_2_ atmosphere.

To explore the effects of bleomycin (MedChem Express, NJ, USA) and Astragaloside IV (AS-IV, Solarbio, Beijing, China) on lipid metabonomics in HUVECs, the cells were treated with bleomycin (50 *μ*M) to induce cell senescence; after which, they were coincubated with AS-IV (50 *μ*M) and bleomycin to explore the effect of AS-IV. At the same time, the effect of different doses of bleomycin on LPC levels in HUVECs was detected. The cells were treated with different concentrations of LPC (0.1 *μ*M, 0.25 *μ*M, and 0.4 *μ*M, Merck Millipore, Burlington, MA, USA) to examine the influence of LPC on cell senescence and damage. To further explore how AS-IV functions in the LPC-induced ferroptosis of endothelial cells, the cells were coincubated with LPC (0.4 *μ*M) and AS-IV (50 *μ*M) with or without FIN56 (Selleck, 5 *μ*M).

### 2.2. LC/MS Metabolomics and Metabolite Identification

HUVECs were divided into the following three treatment groups: (a) control (*n* = 3), (b) bleomycin (*n* = 3), and (c) bleomycin+AS-IV (*n* = 3). Next, the cells were collected and sent to Sensichip Biotech Co., Ltd for use in metabolomic studies. Briefly, the samples (1 × 10^7^ cells) were dissolved in 1500 *μ*L of chloroform/methanol (2/1, *v*/*v* solution), vortexed for 0.5 min, and then dispersed by ultrasonication performed for 30 min at 4°C. Next, 500 *μ*L of water was added to the mixtures, and the mixtures were vortexed for 0.5 min, followed by ultrasonication for 10 min at 4°C. Following sonication, the organic phase was transferred into another EP tube for rotary evaporation. Next, 400 *μ*L of methanol/isopropanol (1/1, *v*/*v* solution) and 5 *μ*L of LPC (12 : 0) internal standard (125 *μ*g/mL) were added to the mixture, which was then kept for 30 min at -20° Celsius. Finally, the mixture was centrifuged at 12,000 rpm for 10 min, and a 200 *μ*L aliquot of the supernatant was removed for use in metabolomic studies that were performed using LC-MS (Thermo Fisher, Ultimate 3000LC, Q Exactive).

The MS parameters used in the study were as follows:

Scan mode: Data Dependent Acquisition (DDA) mode, 1 full scan followed by 10 MS/MS scans. Collision energy was NEC 20; 45 for ion fragmentation. Nitrogen (99.999%) was used as the collision-induced dissociation gas.

Full scan range: 150 to 2000 amu.

Resolution ratio: 70000, AGC: 1e6, IT: 100 ms.

The resolution ratio of data-dependent secondary mass spectrometry: 17500, AGC: 5e5, it: 50 ms.

Spray voltage: 3.8 kV (ESI+), 3 kV (ESI-).

Capillary temperature: 320°C.

S-lens RF level: 50 V.

The Metlin online dataset was used to identify metabolites.

### 2.3. ELISA

For detection of LPC concentrations, the HUVECs were treated with bleomycin and the supernatants were collected for ELISA performed according to instructions provided by the manufacturer. GPX4 activity was detected with a Human GPX4 ELISA Kit. LPC and GPX4 detection kits were purchased from Cloud-clone Corp. (CEK621Ge, Wuhan, China) and Zeye Biotechnology Corp. (ZY-GPX4-Hu, Shanghai, China), respectively.

### 2.4. CCK-8 Assay

HUVECs were added to the wells of 96-well plates (5 × 10^3^ cells per well) and treated as previously described. After being cultured for 24, 48, or 72 h, respectively, CCK-8 solution (10%, Dojindo, Japan) was added to each well and the plates were incubated at 37°C for ~2 h. Finally, the optical density of each well at 450 nm was measured.

### 2.5. TUNEL Staining

HUVECs attached to slides (4 *μ*m) were stained using TdT-mediated dUTP nick end labeling (TUNEL) probes (Merck Millipore), and the cell nuclei were stained with DAPI. Briefly, cells attached to slides were fixed in formalin, washed 3 times with 1x PBS, and then incubated with 0.1% Triton X-100 on ice for 2 min. After being washed again, the cells were incubated with 50 *μ*L of TUNEL staining solution at 37°C for 1 h. Finally, the slides were treated with an antifluorescent quenching sealing liquid and excited at a wavelength of 450~500 nm. Images of the cells were obtained under a fluorescent microscope (Olympus, Tokyo, Japan).

### 2.6. *β*-Galactosidase Staining

HUVECs were seeded into the wells of 24-well plates (3 × 10^4^ cells per well) and treated as previously described. After 72 h, the cells were washed with PBS and fixed with *β*-galactosidase staining stationary solution (Solarbio, China) for 15 min at room temperature. Next, the cells were incubated with staining solution overnight at 37°C, and images of the stained cells were obtained under a microscope (Olympus).

### 2.7. Double Immunofluorescent Labeling

Double immunofluorescent labeling was performed as described in a previous study [22]. Briefly, cells attached to slides were fixed in 4% paraformaldehyde for 24 h at 4°C. After being washed 3 times with PBS, the cells were permeabilized with 0.5% Triton X-100 solution; after which, the cells were incubated for 45 min with the following primary antibodies: rabbit anti-human GPX4 antibody (ab40993; 1 : 100) and mouse anti-human CD34 antibody (ab54203; 1 : 500) (Abcam, Cambridge, MA, USA). Next, the cells were washed 3 more times and then incubated with goat anti-rabbit-FITC labeled secondary antibody and donkey anti-rat-Tex-Red labeled secondary antibody (Abcam). Finally, the fluorescent signals were recorded by a fluorescent microscope (Olympus).

### 2.8. Real-Time PCR

The total RNA in HUVECs was extracted using the TRIzol reagent (Invitrogen, Carlsbad, CA, USA). Next, a 1 *μ*g sample of the total RNA was reverse transcribed into first-strand cDNA using a Reverse Transcription System Kit (Promega, Madison, WI, USA). The cDNA was then amplified and detected by using SYBR Green kits (Thermo Fisher, Waltham, MA, USA) on an Applied Biosystems 7500 StepOne Plus system (Applied Biosystems, Waltham, MA, USA). The primer sequences used in this study were as follows: GPX4 forward 5′-AGAGATCAAAGAGTTCGCCG-3′, reverse 5′-TTGTCGATGAGGAACTGTGG-3′; SLC7S11 forward 5′-GGATTGGCTTCGTCATCACT-3′, reverse 5′-ATAATCAACCCGCGGTACTC-3′; GAPDH forward 5′-TGTTCGTCATGGGTGTGAAC-3′, reverse 5′-ATGGCATGGACTGTGGTCAT-3′. Relative levels of gene expression were calculated using the 2^-*ΔΔ*ct^ method.

### 2.9. Western Blotting

Briefly, the total proteins were extracted by using RAPI lysate solution (Beyotime, Nanjing, China) containing a protease and phosphatase inhibitor cocktail, and the protein concentration in each extract was detected using a BCA kit (Thermo Fisher). Next, the extracted proteins were separated by SDS-PAGE, and the protein bands were transferred onto PVDF membranes. The membranes were then incubated overnight at 4°C with primary antibodies against GXP4 (Abcam, ab125066, 1 : 5000), SLC7A11 (Abcam, ab175186, 1 : 5000), and GAPDH (Abcam, ab181602, 1 : 10000); after which, they were incubated with HRP-conjugated secondary antibodies for 1 h. Finally, the immunostained protein bands were detected using the ECL-Plus reagent (Thermo Fisher) and observed with a Gel Imaging System.

### 2.10. Iron Ion Assay

Intracellular iron ion levels were detected using an iron assay kit (JaICA, CFE-005, Japan). Briefly, the cell lysate was adjusted to a pH of 1.5-3.0 with 6 M HCl and then centrifuged at 6000 rpm for 15 min. The supernatant was collected and used for an iron assay that was performed according to directions provided with the iron assay kit. Absorbance was detected at 560 nm.

### 2.11. Flow Cytometry

HUVECs were treated as previously described. After 72 h, the cells were collected and incubated with a lipid ROS fluorescent probe (C11-BODIPY^581/591^ (D3861, Thermo Fisher)) at 37°C for 1 h. Next, the cells were washed three times with serum-free medium, and the cellular lipid ROS levels were detected by flow cytometry (BD Biosciences, San Jose, CA, USA).

### 2.12. Transmission Electron Microscopy

HUVECs were fixed with a fixative (0.05 M cacodylate buffer containing 2.5% glutaraldehyde and 2% formaldehyde, pH 7.2) for 1 h at room temperature and then overnight at 4°C. After fixation, the samples were immersed in 1% osmium tetroxide (OsO_4_) for 1 h, rinsed with phosphate buffer, dehydrated with different concentrations of ethanol, and then embedded in epoxy resin. Next, an ultramicrotome (Ultracut; Leica, Wetzlar, Germany) was used to obtain 70-80 nm thick sections of the embedded samples which were subsequently stained with uranyl acetate and lead citrate. Finally, the sections were observed using a transmission electron microscope (H-700; Hitachi, Tokyo, Japan) at 80 kV.

### 2.13. Statistical Analysis

All data were processed using GraphPad Prism 7.0 software (GraphPad, San Diego, CA, USA). Differences among multiple groups were compared by one-way ANOVA, and results are presented as a mean value ± standard deviation. A *P* value < 0.05 was considered to be statistically significant. Lipid search software (Thermo) was used to extract and preprocess the LC/MS data of samples. Metabolomic data analyses, including PLS-DA, PCA, and OPLS-DA, were performed using SIMCA-P 13.0 software (Umetrics AB; Umea, Sweden).

## 3. Results

### 3.1. AS-IV Altered the Metabolic Status of HUVECs with Bleomycin-Induced Senescence

**Figure 1:**
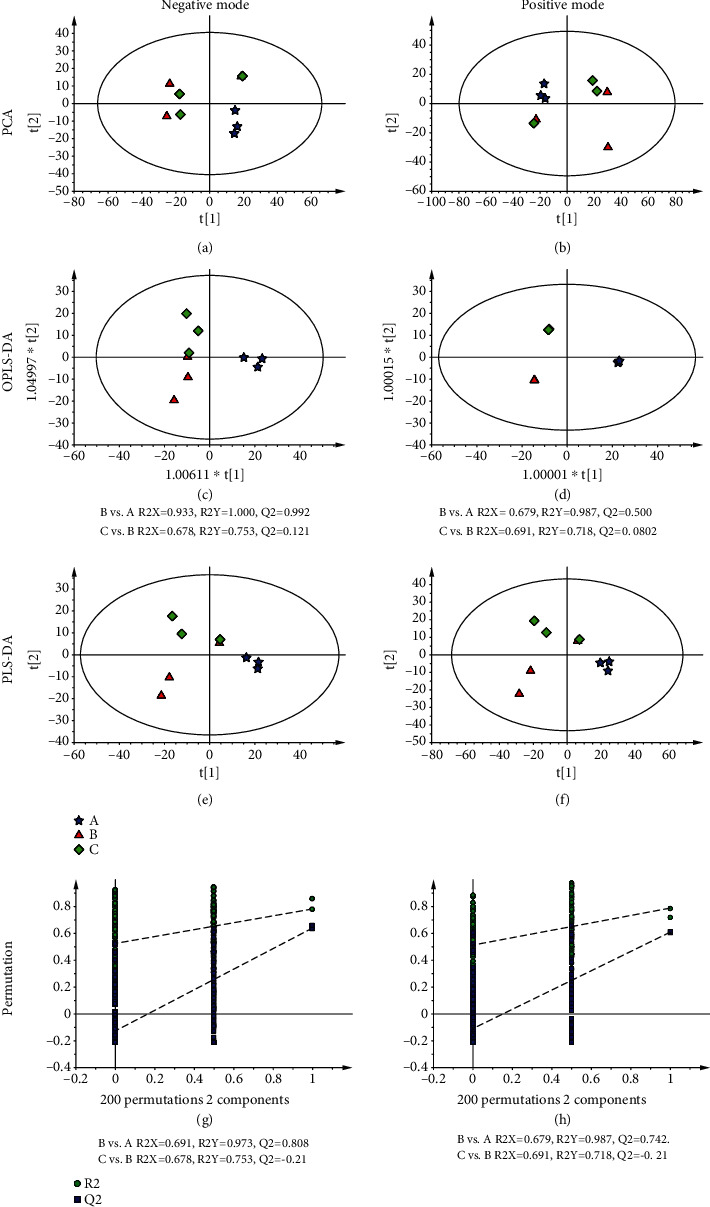
Metabolite profiles in the different groups as analyzed by PCA, PLS-DA, OPLS-DA, and a permutation test performed after metabolomic studies. (a, b) PCA score plots under the negative ion mode and positive ion mode, respectively. (c, d) OPLS-DA score plots under the negative ion mode and positive ion mode, respectively. (e, f) PLS-DA score plots under the negative ion mode and positive ion mode, respectively. (g, h) Permutation tests were performed between groups under the positive ion mode. (e) Model vs. normal. (f) AS-IV treatment vs. model. The letters A, B, and C in the score plots represent the normal group, model group, and AS-IV treatment group, respectively.

To determine whether the metabolites in HUVECs had changed during bleomycin-induced cell senescence and explore the effect of AS-IV on cell senescence, the HUVECs in each group were collected and used for nontargeted metabolomic studies conducted by LC/MS. Chromatograms of samples were obtained under positive and negative modes. Then, the spatial distribution of each cell sample was obtained from principal component analyses (PCAs) that were conducted in the negative and positive ion modes. PCA data showed that the metabolites in each group were well separated. Our results also showed that the metabolites in the model group (B) were significantly different from those in the control group (A) and AS-IV treatment group (C) (Figures 1(a) and 1(b)). In order to obtain additional information about the metabolites that had changed, a OPLS-DA was performed to further analyze the groups. Those results indicated that the model parameters were as follows: *R*^2^*X* = 0.933, *R*^2^*Y* = 1.000, *Q*^2^ = 0.992 (negative ion mode, B vs. A), *R*^2^*X* = 0.678, *R*^2^*Y* = 0.753, *Q*^2^ = 0.121 (negative ion mode, C vs. B), *R*^2^*X* = 0.679, *R*^2^*Y* = 0.987, *Q*^2^ = 0.500 (positive ion mode, B vs. A), and *R*^2^*X* = 0.691, *R*^2^*Y* = 0.718, *Q*^2^ = 0.0802 (positive ion mode, C vs. B) (Figures 1(c) and 1(d)). Given that all the *R*^2^*Y* and *Q*^2^ values were >0.5, we concluded that the PLS-DA model was not overfitted or random. In order to further verify the isolation of samples from the control group, model group, and AS-IV treatment group and identify the marker metabolites, an PLS-DA was performed on each data group. The score plots and permutation test charts are shown in Figures 1(e)–1(h). The parameters were as follows: *R*^2^*X* = 0.691, *R*^2^*Y* = 0.973, *Q*^2^ = 0.808 (negative ion mode, B vs. A), *R*^2^*X* = 0.678, *R*^2^*Y* = 0.753, *Q*^2^ = 0.210 (negative ion mode, C vs. B), *R*^2^*X* = 0.679, *R*^2^*Y* = 0.987, *Q*^2^ = 0.742 (positive ion mode, B vs. A), and *R*^2^*X* = 0.691, *R*^2^*Y* = 0.718, *Q*^2^ = 0.21 (positive ion mode, C vs. B). These results further suggested that our model was reliable (Figures 1(e)–1(h)). All the above data indicated that there was reliable differentiation among the different groups.

### 3.2. Metabolite Profiling and Lipid-Related Pathways

**Figure 2:**
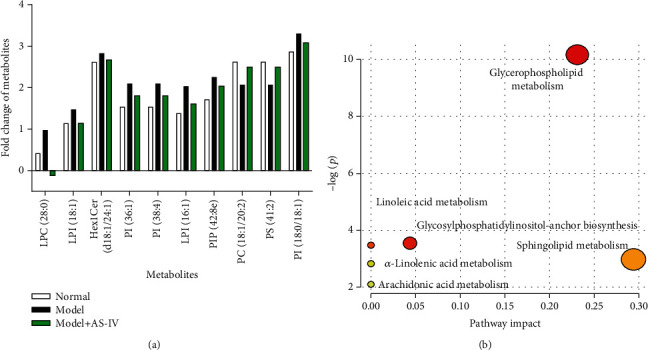
Analysis of different metabolites after metabolomics. (a) The metabolites exhibited in different groups based on metabolomic studies performed under the positive ion mode. Metabolites were obtained using VIP > 1, *P* < 0.05, and error ppm < 10. (b) Plots depict the computed metabolic pathways as a function of −log(*P*) (*y*-axis) and the pathway impacts of the key metabolites (*x*-axis). Metabolites were obtained using VIP > 1 and *P* < 0.05.

A further analysis was performed on the metabolites that were differentially expressed in group B vs. group A and in group C vs. group B. Those data showed that various metabolites including LPC (28:0), LPI (18:1), Hex1Cer (d18:1/24:1), PI (36:1), PI (38:4), PIP (42:8e), LPI (16:1) and PI (18:0/18:1) were upregulated in the bleomycin-induced cell senescence model and downregulated in the AS-IV treatment group, especially LPC (28:0) (Figure 2(a)). Conversely, other metabolites including PS (41:2) and PC (18:1/20:2) showed an opposite trend. Pathway analysis of the differentially expressed metabolites showed that the pathways involved in glycerophosphocholipid metabolism, glycosylphosphatidylinositol metabolism, sphingolipid metabolism, linoleic acid metabolism, *α*-linolenic acid metabolism, and arachidonic acid metabolism were significantly associated with the bleomycin-induced cell senescence model and the effects of AS-IV treatment (Figure 2(b)).

### 3.3. LPC Promoted the Apoptosis and Senescence of HUVECs

**Figure 3:**
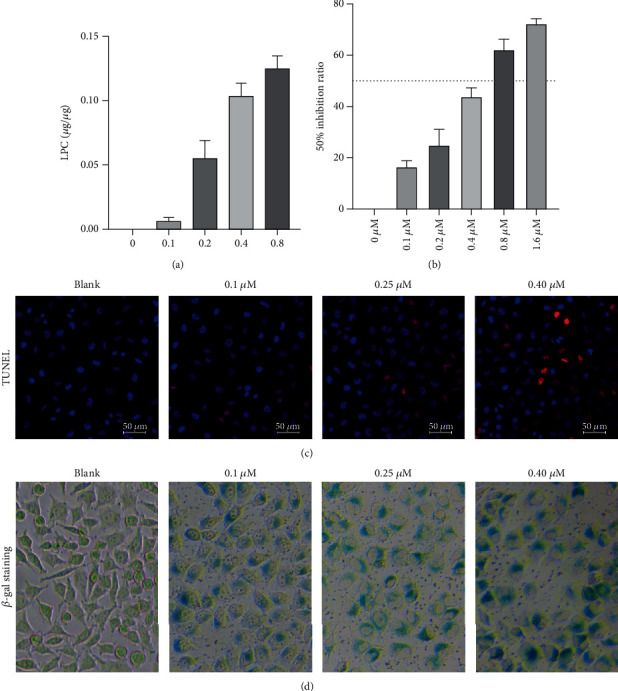
LPC promoted the apoptosis and senescence of HUVECs. (a) The LPC secretion induced by bleomycin was detected by ELISA. (b) The CCK-8 assay was used to detect reductions in cell viability. (c) The effect of LPC on cell apoptosis was observed by TUNEL staining. (d) *β*-Galactosidase staining was performed to detect the effect of LPC on cell senescence.

To further verify that LPC was upregulated in the bleomycin-induced cell senescence model, the HUVECs were treated with bleomycin; after which, ELISA results showed that LPC was markedly upregulated by bleomycin in a dose-dependent manner (Figure 3(a)). Next, the effects of LPC on HUVEC apoptosis and senescence were explored. CCK-8 data indicated that a 0.4 *μ*M concentration of LPC reduced HUVEC viability by 50% (Figure 3(b)). TUNEL and *β*-galactosidase staining results showed that LPC could promote HUVEC apoptosis and senescence in a dose-dependent manner (Figures 3(c) and 3(d)).

### 3.4. AS-IV Attenuated the Effect of LPC on HUVEC Ferroptosis and Senescence

**Figure 4:**
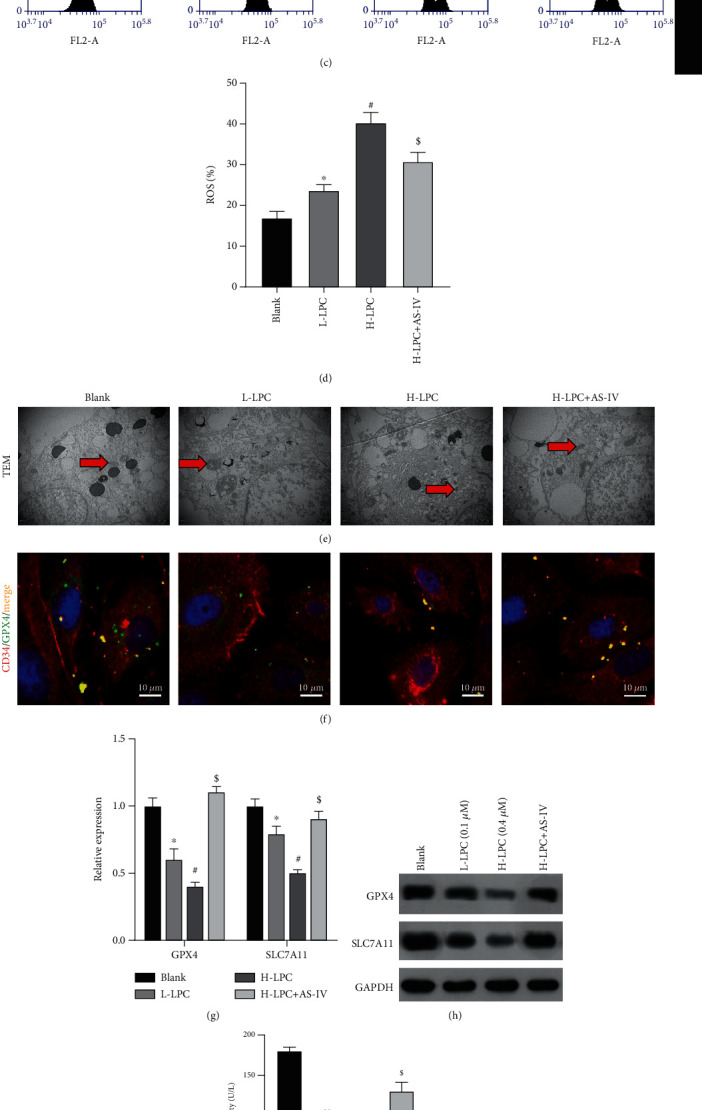
AS-IV attenuated the effect of LPC on HUVEC ferroptosis and senescence. (a–d) The effects of LPC and AS-IV on cell viability, iron ions, and lipid ROS were detected by using the CCK-8 assay (a), an iron assay kit (b), and flow cytometry (c, d), respectively. (e) Transmission electron microscopy was used to observe the morphology of mitochondria in all groups. (f) Immunofluorescence assay of CD34 and GPX4. (g, h) GPX4 and SLC7A11 expression was detected by real-time PCR and Western blotting, respectively. (i) GPX4 activity was detected by ELISA. (j) *β*-Galactosidase staining was performed to detect cell senescence.

To explore the effect of AS-IV on the ferroptosis and senescence induced by LPC in HUVECs, the cells were coincubated with LPC and AS-IV. CCK-8 assay data showed that treatment with AS-IV markedly increased the viability of HUVECs that previously displayed low viability due to treatment with LPC (Figure 4(a)). Moreover, LPC significantly increased the concentrations of iron ions and lipid ROS in HUVECs (Figures 4(b)–4(d)). In contrast, AS-IV treatment dramatically reduced the levels of iron ions and lipid ROS induced by LPC (Figures 4(b)–4(d)). Transmission electron microscopy images showed normal mitochondrial morphology and clear mitochondrial ridges in the control group, while mitochondrial atrophy, decreased or absent mitochondrial ridges, and increased mitochondrial density were observed in both LPC groups, especially in the H-LPC group (Figure 4(e)). Furthermore, AS-IV treatment effectively recovered the mitochondrial morphology that had been altered by LPC (Figure 4(e)). Immunofluorescent assay results showed that GPX4 (green) expression was decreased with increasing LPC treatment, while GPX4 expression was recovered with AS-IV treatment (Figure 4(f)). All these findings indicated that AS-IV could attenuate the ferroptosis induced by LPC in HUVECs. To explore the mechanism by which LPC promotes ferroptosis, expressions of two ferroptosis-related proteins (GPX4 and SLC7A11) were detected. Our data showed that AS-IV treatment could upregulate the levels of GPX4 and SLC7A11 protein and mRNA expression, which had been previously decreased by LPC treatment (Figures 4(g) and 4(h)). Furthermore, AS-IV also increased GPX4 activity, which had been decreased by LPC treatment (Figure 4(i)). These data demonstrated that AS-IV inhibited ferroptosis by upregulating the levels of GPX4 and SLC7A11 expression that were reduced by LPC treatment in HUVECs. In addition, AS-IV decreased the cell senescence induced by LPC treatment in HUVECs (Figure 4(j)).

### 3.5. FIN56 Reversed the Therapeutic Effect of AS-IV on Ferroptosis Induced by LPC Treatment in HUVECs

**Figure 5:**
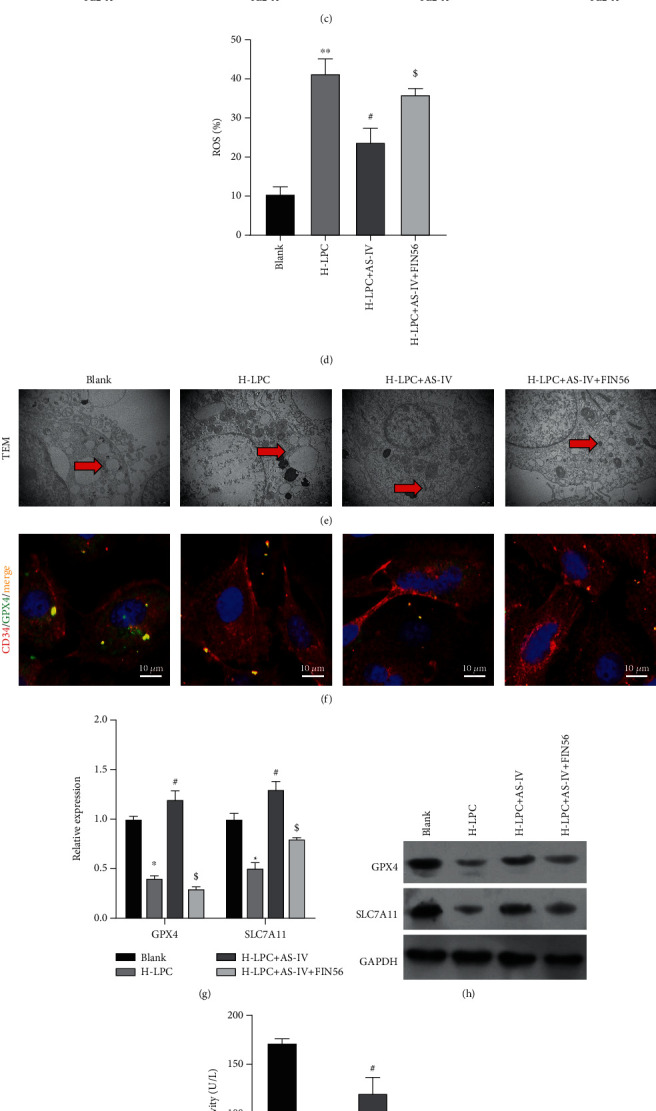
FIN56 reversed the therapeutic effect of AS-IV on the ferroptosis induced by LPC in HUVECs. (a–d) Changes in cell viability, iron ions, and lipid ROS were detected by using the CCK-8 assay (a), an iron assay kit (b), and flow cytometry (c, d), respectively. (e) The morphology of mitochondria in all groups was observed by transmission electron microscopy. (f) Immunofluorescent assay of CD34 and GPX4. (g, h) GPX4 and SLC7A11 expression was detected by real-time PCR and Western blotting, respectively. (i) GPX4 activity was detected by ELISA. (j) *β*-Galactosidase staining was performed to detect cell senescence.

To further verify that the therapeutic effect of AS-IV on HUVECs resulted from inhibition of H-LPC-induced ferroptosis, FIN56 was used as a specific inducer of ferroptosis. CCK-8 assay results indicated that FIN56 could markedly decrease the cell viability that was increased by AS-IV in H-LPC-treated HUVECs (Figure 5(a)). Moreover, the AS-IV-induced decreases in iron ion concentrations and ROS levels in H-LPC-treated HUVECs were both markedly reversed by FIN56 (Figures 5(b)–5(d)). The morphology of mitochondria was observed by transmission electron microscopy. Those observations revealed that the mitochondrial atrophy, incidence of reduced or absent mitochondrial ridges, and the increased mitochondrial membrane density in HUVECs treated with FIN56 were all decreased or absent (Figure 5(e)). These findings indicated that the ability of AS-IV to restore normal mitochondrial morphology in H-LPC-treated HUVECs could be effectively inhibited by FIN56. The immunofluorescent assay showed that GPX4 was suppressed by FIN56, reversing the protective role of AS-IV (Figure 5(f)). Furthermore, the levels of GPX4 and SLC7A11 expression, as well as GPX4 activity, were upregulated by AS-IV in H-LPC-treated HUVECs, and those changes were reversed at both the protein and mRNA levels by FIN56 (Figures 5(g)–5(i)). All these findings indicated that the therapeutic effect of AS-IV on HUVECs was due to the inhibition of the ferroptosis induced by LPC treatment. Additionally, FIN56 induced cell senescence that was reduced by AS-IV in H-LPC-treated HUVECs (Figure 5(j)). The above data showed that FIN56 reversed the therapeutic effect of AS-IV by inducing ferroptosis and cell senescence.

## 4. Discussion

As monolayer cells, vascular endothelial cells cover the surface of the vascular lumen to protect the normal function and structure of the vascular wall. The dysfunction of vascular endothelial cells is often a prerequisite for myocardial ischemia, atherosclerosis, coronary heart disease, and other diseases and also promotes the occurrence and development of various cardiovascular diseases (CVDs) [23–25]. Recently, various studies suggested that endothelial cell dysfunction might also be caused by a new mode of cell death (ferroptosis) in addition to apoptosis/necrosis and autophagy [14]. However, because the existing research on ferroptosis is in its early stage, the related roles and mechanisms of ferroptosis require further exploration. Because bleomycin is known to induce apoptosis and senescence in epithelial and nonepithelial cells in the lung [26], we used bleomycin with/without AS-IV to treat endothelial cells and selected LPC as a lipid metabolite that undergoes one of the most significant changes in expression. We then showed that AS-IV protects the function of endothelial cells by inhibiting ferroptosis.

As the main component of oxidized low-density lipoproteins (ox-LDLs), LPC plays a pivotal role in promoting the development of diseases, and especially cardiovascular diseases, by inducing oxidative stress and promoting cell apoptosis [27]. However, few studies have examined the relationship between LPC and ferroptosis. Meanwhile, a recent study by Bai et al. [28] showed that after ox-LDL treatment, the influx of iron into aortic endothelial cells increased, ROS levels also significantly increased, and cell death occurred. However, the phenomenon of cell death was significantly reversed by the use of ferroptosis inhibitors. Yang et al. [29] found that ox-LDLs induce ferroptosis in HCAECs by promoting activation of the Nrf2 pathway [29]. In this study, we found that LPC treatment could cause ferroptosis of human umbilical vein endothelial cells by inducing cell apoptosis, senescence, and ROS production.

With increasing age, the proliferation and migration abilities of vascular endothelial cells decrease, cellular oxidation products accumulate, cell permeability changes, and cell phenotype transformation and cell apoptosis occur, all of which can lead to vascular senescence that finally induces the occurrence and development of cardiovascular diseases, such as atherosclerosis [30]. Although researchers have found that RAAS, oxidative stress, sirtuins, and autophagy are closely related to vascular aging [9, 31–33], the role played by ferroptosis in vascular aging, as well as the effect of AS-IV on the ferroptosis of vascular endothelial cells, has not been reported. Here, we showed that AS-IV can protect the function of endothelial cells by inhibiting ferroptosis, suggesting that AS-IV might to some extent prevent vascular aging and reduce the occurrence and development of cardiovascular disease.

In this study, we used bleomycin to induce senescence in HUVECs and then treated the cells with AS-IV to identify and select the differential metabolites. After a lipid metabolomic analysis, LPC was selected as the research molecule. LPC, as the main active component of ox-LDLs, often damages and causes the dysfunction of vascular endothelial cells [34]. A previous study suggested that ox-LDLs can induce cell senescence in endothelial cells [35]. Studies showed that LPCs can induce ROS production by activating P38 and NF-*κ*B, can also enhance the production of superoxides by activating endothelial NADPH oxidase, and thereby cause the apoptosis of vascular endothelial cells [36, 37]. Consistent with those findings, we showed that cell viability was greatly suppressed, while cell apoptosis and senescence were significantly promoted under conditions of LPC treatment. Furthermore, ROS levels were upregulated with an increase in LPC treatment. All these results indicated that LPC inhibited the proliferation and promoted the apoptosis and senescence of endothelial cells.

Previous studies proved that AS-IV protects endothelial function by reducing ROS production and oxidative stress [20, 38]. Here, we also demonstrated that the levels of LPC-induced ROS in HUVEC cells were obviously downregulated by AS-IV treatment, while that trend was greatly reversed by treatment with a ferroptosis agonist (FIN56), indicating that AS-IV inhibited ferroptosis by reducing ROS production. The main source of ROS in endothelial cells is the nicotinamide adenine dinucleotide oxidase (NADPH oxidase) system [39]. Xu et al. found that AS-IV can protect HUVECs from H_2_O_2_-induced oxidative stress by inhibiting the NADPH oxidase-ROS-NF-*κ*B pathway and eNOS uncoupling. Therefore, we speculated that AS-IV might reduce ferroptosis in HUVEC cells, partially by inhibiting the NADPH oxidase-ROS-NF-*κ*B pathway and eNOS uncoupling.

Given that Fe^2+^/Fe3^+^, one of the key causes of ferroptosis, participates in ROS formation via both enzymatic and nonenzymatic reactions [40], we also showed that AS-IV can significantly suppress the production of LPC-induced iron ions, while a ferroptosis agonist (FIN56) largely reversed that phenomenon. This further confirmed that AS-IV can protect against ferroptosis by downregulating free iron concentrations and reducing ROS production. We also found that AS-IV regulates ferroptosis in endothelial cells by promoting SLC7A11 and GPX4 expression, and the ferroptosis agonist FIN56 can suppress the upregulation of those molecules. System Xc is composed of SLC7A11 and solute carrier family 3 member 2 (SLC3A2) [41]. SLC7A11 is the main active subunit of the reverse transporter, which regulates the dynamic balance of intracellular GSH [42]. Extracellular cystine extracted by System Xc is often used to synthesize glutathione [38]. GPX4, as a central regulator of ferroptosis, converts reduced glutathione to oxidized glutathione and removes intracellular lipid ROS [43]. We demonstrated that AS-IV can inhibit ferroptosis by regulating SLC7A11 and GPX4 expression and thereby help protect endothelial cells. Besides the cystine/glutamate antiporter, the System Xc pathway, other signaling pathways involved in ferroptosis include the iron homeostasis regulatory pathway, voltage-dependent anion channel (VDAC) pathway, pentose phosphate pathway, hydroxyvalerate pathway, and AMP-activated protein kinase- (AMPK-) BECN1 pathway. All those signaling pathways intersect with each other to exert a comprehensive effect [11, 41, 44, 45]. However, we did not thoroughly investigate the mechanism by which AS-IV plays its role in the LPC-triggered ferroptosis of endothelial cells, and that mechanism requires further study. While previous studies proved that GPX4 is the main molecule involved in ferroptosis, it was recently found that FSP1, as a negative regulatory factor, is also involved in ferroptosis [46]. The effect of AS-IV on FSP1 expression will be investigated by us in the near future.

With regard to morphology, ferroptosis mainly manifests as pyknosis of the mitochondrial membrane; increased membrane density; blurred, reduced, or absent mitochondrial cristae; and intact nuclear membranes [47]. Here, we found that the mitochondria in endothelial cells underwent morphological change characteristic of ferroptosis during treatment with LPC, but those changes were greatly relieved by AS-IV treatment, which further confirmed the role of AS-IV in ferroptosis.

An excessive accumulation of iron ions in aging cells can lead to DNA damage, inhibit DNA repair, and thus accelerate the process of aging, which is defined as ferrosenescence [48]. Studies showed that iron can directly cause DNA damage in endothelial cells, and the damage process begins within minutes of iron ingestion [49]. Therefore, we used bleomycin, an important chemotherapeutic drug, which is reported to induce senescence in vascular smooth muscle cells, alveolar epithelial cells, and other cells [3, 50], and found that cell senescence was enhanced by LPC treatment but alleviated by AS-IV treatment, indicating that AS-IV can reduce LPC-induced ferrosenescence in endothelial cells.

## 5. Conclusion

In conclusion, this study demonstrated that AS-IV helps to protect against the ferroptosis of endothelial cells. As a differential lipid metabolite of AS-IV in bleomycin-treated endothelial cells, LPC was identified by performing a lipid metabolomic analysis. The LPC-suppressed proliferation and LPC-induced apoptosis and senescence of endothelial cells were greatly attenuated by AS-IV treatment, but treatment with a ferroptosis agonist (FIN56) largely reversed those trends. All these results suggest that AS-IV could be used as a new drug for treating ferroptosis-related diseases.

## Data Availability

All data generated or analyzed in this study are available in the published article.

## Additional Points

*Highlights*. (1) AS-IV altered the metabolic status of HUVECs with bleomycin-induced cell senescence. (2) LPC promoted lipid peroxidation-mediated cell death and senescence in HUVECs. (3) AS-IV attenuated the ferroptosis and senescence induced by LPC in HUVECs.
